# Initial Clinical Experience with NIPT for Rare Autosomal Aneuploidies and Large Copy Number Variations

**DOI:** 10.3390/jcm11020372

**Published:** 2022-01-13

**Authors:** Thomas Harasim, Teresa Neuhann, Anne Behnecke, Miriam Stampfer, Elke Holinski-Feder, Angela Abicht

**Affiliations:** Medical Genetics Center, Bayerstrasse 3-5, D-80335 Munich, Germany; Teresa.Neuhann@mgz-muenchen.de (T.N.); Anne.Behnecke@mgz-muenchen.de (A.B.); Miriam.Stampfer@mgz-muenchen.de (M.S.); Elke.Holinski-Feder@mgz-muenchen.de (E.H.-F.); Angela.Abicht@mgz-muenchen.de (A.A.)

**Keywords:** non-invasive prenatal testing, rare autosomal aneuploidies, copy number variations, clinical significance

## Abstract

Objective: Amniocentesis, chorionic villi sampling and first trimester combined testing are able to screen for common trisomies 13, 18, and 21 and other atypical chromosomal anomalies (ACA). The most frequent atypical aberrations reported are rare autosomal aneuploidies (RAA) and copy number variations (CNV), which are deletions or duplications of various sizes. We evaluated the clinical outcome of non-invasive prenatal testing (NIPT) results positive for RAA and large CNVs to determine the clinical significance of these abnormal results. Methods: Genome-wide NIPT was performed on 3664 eligible patient samples at a single genetics center. For patients with positive NIPT reports, the prescribing physician was asked retrospectively to provide clinical follow-up information using a standardized questionnaire. Results: RAAs and CNVs (>7 Mb) were detected in 0.5%, and 0.2% of tested cases, respectively. Follow up on pregnancies with an NIPT-positive result for RAA revealed signs of placental insufficiency or intra-uterine death in 50% of the cases and normal outcome at the time of birth in the other 50% of cases. We showed that CNV testing by NIPT allows for the detection of unbalanced translocations and relevant maternal health conditions. Conclusion: NIPT for aneuploidies of all autosomes and large CNVs of at least 7 Mb has a low “non-reportable”-rate (<0.2%) and allows the detection of additional conditions of clinical significance.

## 1. Introduction

Chorionic villi sampling (CVS) or amniocentesis (AC) allow a definitive diagnosis of any aneuploidy or large CNVs by conventional karyotyping. Molecular karyotyping by chromosomal microarray (CMA) allows the detection of any aneuploidy and CNVs of large (e.g., >10 Mb) or small size (e.g., so-called micro-deletions or duplications) [1]. CfDNA-based NIPT was introduced with an initial focus on common trisomies (13, 18, and 21) exhibiting sensitivities between 95.8–99.7% and specificities in the range of 99.8%–99.9% [2,3]. Nevertheless, it has been demonstrated that structural ultrasound abnormalities and serum marker levels (below 0.2 multiples of the median (MoM)) detected during first trimester combined testing (FCT) can point to other atypical chromosomal anomalies (ACA), namely rare autosomal aneuploidies (RAA) and CNVs. These anomalies can be clinically significant and would have been missed by NIPT focused on common trisomies exclusively [4,5]. The clinical significance and prevalence of ACA as diagnosed by classic karyotyping are outlined by combined data from 16 population-based congenital anomaly registries [6]. Within 10,323 analyzed cases with chromosomal abnormality, 2% had RAA. Of these, all non-mosaic RAAs (29%) did not proceed to term, as expected. In contrast, the mosaic RAAs (70%) resulted in 58 liveborn babies, 10 stillbirths, and 69 terminations of pregnancies (TOP) due to fetal anomaly. CNVs were found in 5.8% of pregnancies. Unbalanced chromosomal translocations accounted for 2.1% of all CNV-positive cases. In sum, the prevalence of large ACAs detectable by classic karyotyping and, therefore, also by NIPT in these 16 registries was conservatively estimated to be at least 7.5%, exceeding that of trisomy 13 and underlining their clinical relevance.

With the advent of genome-wide NIPT, test options have expanded to include ACA. As a result, new data about their clinical significance started to emerge. The majority of all cases positive for RAA were associated with negative pregnancy outcome, namely intra-uterine growth restriction (IUGR), miscarriage, stillbirth, true fetal mosaicism, congenital abnormalities, and confirmed or suspected uniparental disomy [7,8,9,10,11,12]. The remaining pregnancies resulted in a normal clinical outcome with a normal fetal karyotype. As most of the RAAs were determined to be confined to the placenta, the degree of placental mosaicism, the kind of nondisjunction (meiotic or mitotic error) and the aneuploid chromosome itself were suspected to be the main variables for clinical outcome prediction. As a result, a NIPT screening strategy with focus on the most clinically significant RAAs was proposed [13]. Similarly, around 50% of CNVs detected by NIPT were confirmed in the fetus and associated with a negative clinical outcome; some (around 6%) were confirmed in the mother while the rest remained unconfirmed [7,10,12,14,15,16,17,18,19,20]. Recent publications evaluated the positive predictive values (PPVs) for ACA in high risk- and mixed-risk populations. In the former population, the PPV was 15% compared to 6% in the mixed risk population [10,16]. 

The current study’s objective was to determine retrospectively the clinical outcome of patients who received a positive NIPT result by a pre-validated genome-wide NIPT method (VeriSeq NIPT solution v2, Illumina, Inc., San Diego, CA, USA). While the scope of testing included RAAs and large CNVs in addition to common aneuploidies, we focused on the clinical implications of ACAs.

## 2. Materials and Methods

### 2.1. Samples and Testing

This descriptive study was approved by the MGZ Institutional Review Board (approval #00108) in May 2019. Overall, 3664 unselected patients were referred to the Medical Genetics Center for NIPT between October 2019 and September 2020. Inclusion criteria for NIPT were a minimal gestational age of 9 + 0 weeks, singleton or (vanishing) twin pregnancies and completed a priori genetic counseling. All patients gave written informed consent for NIPT, complying with the German Genetic Diagnostic Act (GenDG), and usage of their data and remaining sample material for quality management and scientific purposes.

The test request form includes fields to indicate whether the patient is currently using low molecular weight heparin as well as the maternal height, weight, and test indications. Pre-defined indications comprised advanced maternal age (≥35 years at the estimated date of delivery), increased adjusted risk for aneuploidies determined by FCT, structural abnormalities detected by ultrasound, increased genetic risk (e.g., chromosomal translocations), or other medical reasons to be specified. 

NIPT was offered initially with three test options: test option 1 (for trisomy 21 exclusively), test option 2 (for common trisomies 13, 18, and 21), and test option 3 (for common trisomies and sex chromosomal aneuploidies (SCAs)). For test options 2 and 3, an add-on for RAA and CNVs (>7 Mb) was available (Table 1). Sex chromosomal aberrations are not reported for twin pregnancy samples. For all test options, fetal sex reporting was optional. 

### 2.2. Clinical Follow-Up

Clinical follow-up information was requested retrospectively by a standardized questionnaire sent to each individual physician who received a positive test report for either common trisomies, SCAs, or ACAs. The results of CVS, AC, ultrasound examination, or other diagnostic interventions were inquired. The outcome of pregnancy could be indicated as follows: (1) proceeded to term, (2) intra-uterine fetal death, and (3) induced abortion. 

### 2.3. Test Method

The CE-IVD certified NIPT, Veriseq NIPT Solution v2 (Illumina, Inc., San Diego, CA, USA), was performed as instructed by the product insert and as previously described. Fetal fraction was estimated from the normalized sequencing data by integration of fragment length information and quantification of observed sequencing depth in fetal fraction predictive regions as described in [21]. A quality control step calculated the iFACT value (individualized fetal aneuploidy confidence test); this value depicts a ratio of the fetal fraction and the observed sequencing coverage and judges if either one or both parameters are high enough for a confident call on aneuploidies and imbalances [21]. Therefore, a dynamic instead of a fixed fetal fraction cutoff is applied. The term “mosaic” in this study refers to placental mosaicism based on NGS-NIPT results. The bioinformatic pipeline calculates a mosaic ratio for each chromosome tested by using the overall fetal fraction (see above) and the so called affected fetal fraction (fetal fraction based on the number of surplus reads of the chromosome tested). As both kinds of fetal fraction quantifications are susceptible to measurement errors, the mosaic ratio allows only an approximate classification as low-, middle-, high-grade mosaic, or non-mosaic status of the chromosome tested. The test procedure was validated a priori with 234 verification samples. In rare cases with borderline results or test failures, the sample was reanalyzed using residual plasma from the original blood sample. If the reanalysis also failed, a new blood sample was requested without specifying any waiting time for the second withdrawal. In case of low molecular weight heparin medication of the patient, a temporal maximization between last heparin injection and subsequent NIPT blood withdrawal was requested. If the second blood draw also failed, no further testing was performed, and a report was generated stating that no calls could be made due to repeated test failures for unknown reasons. 

### 2.4. Data Analysis 

The software package Excel 2013 (Microsoft, Redmond, WA, USA) was used for the collection and analysis of anonymized follow-up data reported back to us. Patient demographics were calculated, where available, based on the information on the test request form. All information received was assumed to be correct. Test performance metrics were extracted from the laboratory information management system. The turnaround time for NIPT was calculated as the interval between the day the sample arrived in the lab and the day of medical validation.

## 3. Results

### 3.1. Patient Demographics and Test Metrics

Patient demographics are presented in Table 1. The tested patients represent an obstetric population with a mixed risk profile (mean maternal age 34.3 ± 4.7 years). Where indicated on the test request form, patients requested NIPT mainly due to an advanced maternal age (≥35 years; 46.9%) or maternal concern about fetal wellbeing (maternal indication; 44.6%). Where patients selected testing for all autosomes and CNVs, neither a history of recurrent pregnancy loss, growth retardation, placenta insufficiency, nor a family history of CNVs was mentioned in terms of testing indications. Results were reported on average in around 3–5 working days (Table 2). The test failure rate accounted for 1.1% overall (42 out of 3664 tests), comprising mostly samples that failed after the initial analysis but that could be analyzed successfully after repeated testing of the same blood tube (1.0%). Only 5 (0.14%) samples remained unresolved after analysis of a newly withdrawn blood sample: the corresponding physicians were informed about the expected low success rate of a test repetition for the specific patient and that diagnostic alternatives should be considered. Reasons for failure were low fetal fraction or low sequencing coverage, or both (iFACT failure). The mean coverage per sample was 9.0 million paired-end reads (± 3.4 million reads). 

### 3.2. Overall NIPT Results 

**Figure 1:**
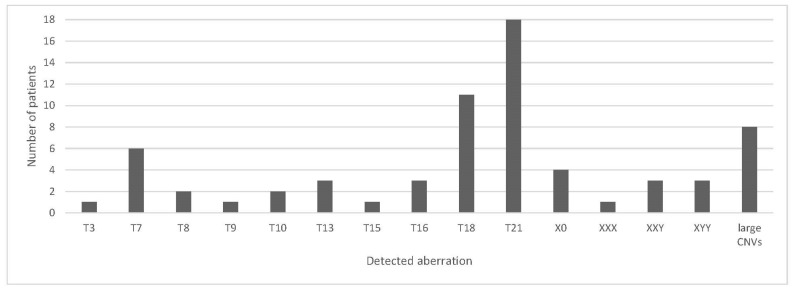
Detected chromosomal aberrations in the study population. Trisomy 21 was the most common trisomy detected, followed by trisomy 18. Concerning sex chromosomal aneuploidies (SCAs) and rare autosomal aneuploidies (RAA), XXY and trisomy 7 had the highest frequency.

Overall, 67 out of 3664 unique samples (1.8%) were classified as high risk for chromosomal aberrations, with 32 common trisomies (0.9%), 11 SCAs (0.3%), 16 RAAs (0.5%) and 8 CNV cases (0.2%) (Figure 1). Although outcome information was only solicited systematically in high-risk result patients, one false-negative test result was reported in a dichorionic twin pregnancy where one fetus was diagnosed with trisomy 21 after receiving a negative NIPT result (estimated fetal fraction 4%, 11.2 million reads). 

### 3.3. Common Autosomal and Sex Chromosomal Aneuploidies

Clinical follow-up information was available for 14 cases positive for trisomy 21, 8 cases positive for trisomy 18, 2 cases positive for trisomy 13, and 5 cases positive for SCA (2x XYY, 2x monosomy X, 1x XXY; Table 3 and Table 4).

### 3.4. Atypical Chromosomal Aneuploidies (ACAs)

**Figure 2:**
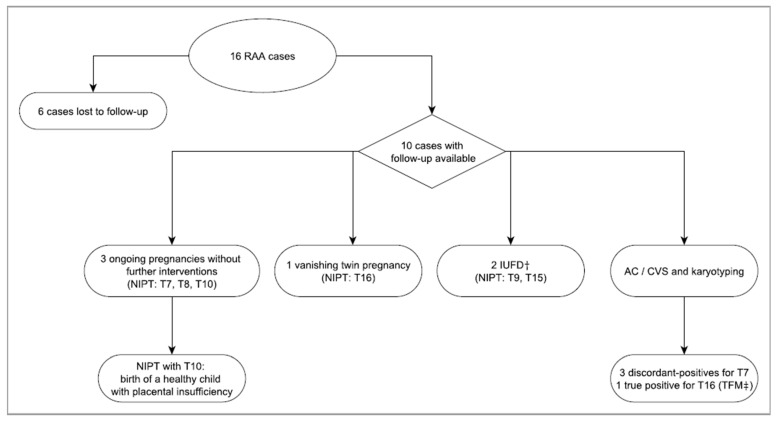
Flowchart of RAA positive cases and their clinical outcome. For 10 cases clinical outcome information was available. All NIPT results indicating the presence of a non-mosaic RAA had an adverse pregnancy outcome (T9, T10, T15, T16). The other RAAs presented in a mosaic form. ^†^ intra-uterine fetal death, ^‡^ true fetal mosaicism.

There were 16 cases with an NIPT result indicating a high risk for an RAA; clinical follow-up information was available for 10 cases (Figure 2 and Table 5). Based on the mosaic ratio on the NIPT sequencing data, four were classified as non-mosaic aneuploidies and 12 cases were considered to be mosaic. The four NIPT cases classified as non-mosaic RAA included a trisomy 9, a trisomy 10, a trisomy 15, and a trisomy 16. The trisomy 9 and 15 cases resulted in intra-uterine fetal death (IUFD). In the trisomy 10 case, signs of placental insufficiency were reported. Neither prenatal nor postnatal diagnostic procedures were performed; the child was born apparently healthy after clinical examination. NIPT performed for a pregnancy with sonographic anomalies reported non-mosaic trisomy 16. At amniocentesis, trisomy 16 was detected in 10% of amniotic cells. In view of the ultrasound findings, UPD16 and 22q11.2 deletion syndrome were also tested for, and the results were normal. In summary, out of four non-mosaic RAA cases, one was confirmed in fetal tissue; in the other three, no confirmatory testing was done. All four pregnancies had adverse pregnancy outcomes. 

The 12 NIPT results classified as mosaic RAA included trisomy 3 (*n* = 1), trisomy 7 (*n* = 6), trisomy 8 (*n* = 2), and trisomy 16 (*n* = 1). The trisomy 3 case was lost to follow-up. Of the six mosaic trisomy 7 reports, four had follow-up information, of which three were not confirmed by AC and the fourth resulted in a live-born child with normal features at clinical examination. A low-grade mosaic trisomy 8 (~30%) did not reveal abnormalities by follow-up ultrasound performed at the 17th week of gestation; patient was lost to follow-up. The initial NIPT result of a mosaic trisomy 16 in a twin pregnancy was followed by a vanishing twin event. A second NIPT, performed 6 weeks after the vanishing event, was normal, indicating that the trisomy 16 was highly likely the cause of the vanishing twin event. Thus, only one of the mosaic RAAs (mosaic trisomy 16 and vanishing twin) was confirmed to have clinical significance. 

### 3.5. Copy Number Variations (CNV) 

**Figure 3:**
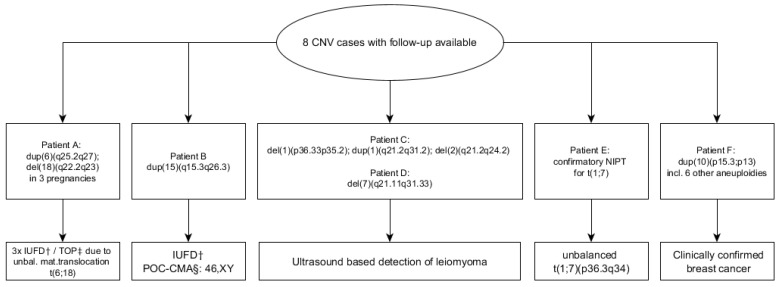
Flowchart of CNV-positive cases and their clinical outcome. CNVs detected by NIPT had a size ranging from 9–58 megabases. The NIPT result from patient F presented a combination of a non-mosaic duplication on chromosome 10 and six RAAs in a mosaic form. ^†^ intra-uterine fetal death; ^‡^ termination of pregnancy; ^§^ analysis of the product of conception by chromosome microarray.

CNVs with imbalances of 9–58 Mb in size were detected in eight cases, and follow-up results were available for all of these patients (Figure 3). The first patient, Patient A (Figure 3), a 35-year-old mother of a healthy 9-month-old son, had genome-wide NIPT at a gestational age of 9 + 6 weeks. NIPT revealed a 17 Mb duplication on chromosome 6 and a 9.5 Mb deletion on chromosome 18 (dup(6)(q25.2q27);del(18)(q22.2q23)). The fetal fraction was 8% and 9.7 million reads were analyzed. Both imbalances were localized close to the telomeres, which prompted the supervising genetic counselor to suspect a parental balanced reciprocal translocation. Shortly after post-test genetic counseling, ultrasound examination revealed major fetal malformations. Subsequently, the patient was diagnosed with a missed abortion, which unfortunately was not karyotyped. Parental karyotyping confirmed a maternal, balanced reciprocal translocation (46,XX,t(6;18)(q25.2;q22.2)) concordant with the chromosomal breakpoints reported by NIPT. Two additional pregnancies of the couple resulted in an identical NIPT result (case 2 and 3) followed by confirmatory karyotyping and subsequent induced abortion. The fourth case received a triple imbalance NIPT result with two deletions and one duplication (patient C in Figure 3). Interestingly, one deletion in chromosome 1p36.33 was reported in the context of NIPT confounding uterine leiomyoma (UL) [22]. High-resolution ultrasound examination revealed a highly vascularized uterine mass, most likely a UL. No further follow-up was available. In the fifth case, a UL of significant size was detected after NIPT had reported a 49 Mb deletion on chromosome 7 (del(7)(q21.11q31.33)) (patient D in Figure 3). A follow-up ultrasound examination of the fetus did not show any abnormalities. In the sixth case, a low-grade mosaic duplication on chromosome 15q15.3–15q26.3 (~30%) was detected by NIPT (patient B in Figure 3). This pregnancy resulted in IUFD, and cells from the product of conception (POC) had a normal karyotype revealed by chromosomal microarray analysis. In the seventh case, NIPT was used in a woman with a balanced reciprocal translocation t(1;7)(p36.3;q34) (patient E in Figure 3). A duplication of 20 Mb was identified mapping to chromosome 7q34–36.3, confirming an unbalanced karyotype, which was detected by amniocentesis performed simultaneously. The eighth case, Patient F (Figure 3), was a 23-year-old patient with a monochorionic twin pregnancy. At 22 + 4 weeks of gestation, ultrasonography revealed a ductus venosus agenesis in one fetus. NIPT was performed and showed a non-mosaic 16 Mb duplication on chromosome 10 and six low-grade (<20%) mosaic aneuploidies (report result: −4;−5;+6;dup(10)(p15.3p13);+14;+18;+21). The fetal fraction was 18% and the sequencing depth 15.8 million reads. Medical follow-up revealed an aggressive form of breast cancer. 

## 4. Discussion

This NIPT study was primarily focused on the clinical outcome of positive results and the clinical significance of RAA and CNVs. Based on additional available follow-up information, clinical outcome data for the common trisomies and SCAs were consistent with the published test performance of the manufacturer [23]. The clinical follow-up for cases where NIPT indicated a RAA or CNV reported here added to the limited data available in the published literature.

Analysis on a per chromosome basis revealed a distinct outcome pattern of RAAs. All of the trisomy 7 cases of this study showed low rates of mosaicism, and those with available clinical data all had a normal fetal outcome. This observation is in line with similar NIPT data showing trisomy 7 as the most frequent RAA in the absence of fetal involvement [13]. The absence of fetal anomalies can be explained by the observed confinement of this particular mosaic trisomy to the cytotrophoblast representing confined placental mosaicism type 1 [24]. This type of CPM most likely is a result from a mitotic non-disjunction event, as these errors usually involve only one embryonic cell lineage and are associated, as observed, with low rates of mosaicism [25,26]. Nevertheless, mosaic trisomy 7 has been associated with fetal growth restriction, and fetal growth monitoring, therefore, is recommended [9]. In contrast, we detected a trisomy 10 in a non-mosaic form that resulted in placental insufficiency with a clinically apparently healthy child born. This can be explained by an initial trisomic conceptus that experienced a mitotic rescue event after compartmentalization of the embryonic and extra-embryonic tissue [27]. Trisomy 16 is assumed to be responsible for one vanishing twin event and was associated with structural aberrations and true fetal mosaicism in another case. A PPV value for an adverse pregnancy outcome for this trisomy based on meta-analytical literature accounts for 65% [28]: characteristic phenotypes for a mosaic trisomy 16 seem to be low birth weights and preterm births [29]. Importantly, it has been shown that when trisomy 16 is restricted exclusively to the cytotrophoblast and amniotic cells have normal karyotypes, abnormal pregnancy outcomes can be observed [30]. Similarly, the PPV for adverse outcomes (fetal loss, true fetal mosaicism, and syndromes caused by uniparental disomy 15) with trisomy 15 is reported to be >95%. This is consistent with the non-mosaic trisomy 15 NIPT case in this study, which resulted in IUFD. Taken together, the mosaic RAAs of this study population had a favorable outcome, while the non-mosaic aneuploidies had adverse outcomes. Although, this pattern is also observed with common trisomies [31], it has to be interpreted in the context of this study´s small size and its strong bias towards non-mosaic trisomies with a known association for adverse pregnancy outcome [13].

Our results support previous work showing that NIPT detection of CNVs has clinical value that may extend beyond the detection of fetal anomalies. Here, NIPT proved its capability to repeatedly detect unbalanced forms of reciprocal translocations from two maternal balanced reciprocal translocation carriers. This is in line with a retrospective study showing a sensitivity and specificity of nearly 100% for unbalanced translocation detection [32]. This high sensitivity was obtained for a specific minimal size of one translocated CNV of 15 megabases and an average sequencing depth of about 11 million single-end reads of the corresponding NIPT, both critical parameters for CNV sensitivity. Additionally, three suspected false-positive NIPT results were indicative of confounding maternal conditions such as breast cancer and benign UL. UL is a common condition with a suspected occurrence of up to 25% among women above 30 years of age [33]. Genomic profiling of UL-specific DNA revealed several CNVs over a broad range of chromosomes [34,35]. Dharajiya et al. reported on NIPT results from 20 patients with clinically confirmed leiomyoma: CNVs detected in plasma cfDNA by NIPT were concordant to CNVs detected in leiomyoma biopsies analyzed by SNP array in the same patient, providing proof for the leiomyomatous origin of these imbalances [36]. Interestingly, some detected deletions in patients C and D of this study (del(1)(p36.33p35.2) and del(7)(q21.11q31.33)) were described previously in the context of circulating tumor DNA (ctDNA) based detection of UL and leiomyosarcoma, pointing towards a common CNV-specific etiology of these entities [37].

The result of a clinically confirmed breast cancer, as detected in patient F, presented with a CNV in concert with multiples aneuploidies. Multiple aneuploidies, defined as more than one aneuploidy and unique and non-specific CNVs, have been described as incidental NIPT results pointing towards occult maternal malignancies [38,39]. The isolated CNV of patient F seems to be atypical for breast cancer, since the majority of patients show several CNVs on multiple chromosomes [40,41]. Due to the size limitations for a positive CNV call with the NIPT method used, it is possible that other CNVs remained undetected due to a smaller size below 7 megabases. Cancer detection per se by NIPT lacks performance metrics, and in case of breast cancer specifically, depends largely on the tumor stage and immunophenotype [40]. Taken together, the details of this case provided proof-of-principle for NIPT serving as a liquid biopsy screening method for neoplasia, although further studies are necessary. Pre-test counseling should include the possibility of such incidental findings and determine the patient´s choice regarding disclosure of such results. 

The main limitation of this study is its limited and biased follow-up, since only positive NIPT cases were investigated. A performance evaluation in terms of observed sensitivity is not possible with the data set available, since true aberrations not detected by NIPT were not reported. Additionally, the clinical implications of the detected imbalances are based exclusively on feedback from different corresponding physicians. The described adverse clinical outcome may not be a direct result of the aberrations detected by NIPT. On the other hand, AC or CVS results might be false-negative due to a low amount of sample material, a non-representative character of the sample, or due to limited resolution of the karyotyping method performed. Authors should discuss the results and how they can be interpreted from the perspective of previous studies and of the working hypotheses. The findings and their implications should be discussed in the broadest context possible. Future research directions may also be highlighted.

## Figures and Tables

**Table 1:** Patient demographics of the study population.

Variable	*n* = 3664 ^†^
**Maternal age (years)**	
*n*	3664
Mean ± Standard deviation (SD)	34.3 ± 4.7
Min-max	17.0–60.0
**Gestational age (weeks)**	
*n*	3664
Mean ± SD	13 ± 2
Min-max	10–32
**Gestational age group, *n* (%)**	
*n*	3664 (100)
1. trimester (10–13.9 weeks) ^‡^	2223 (60.6)
2. trimester (14–27.9 weeks)	1438 (39.2)
3. trimester (28–40+ weeks)	3 (0.1)
**Test indication, *n* (%)**	
*n*	3081 (100)
Advanced maternal age	1446 (46.9)
maternal indication ^§^	1374 (44.6)
increased adjusted trisomy risk after FCT ^¶^	148 (4.8)
Ultrasound abnormalities	98 (3.2)
increased genetic risk for aneuploidy	15 (0.5)
**Body mass index (BMI); *n* (%)**	
*n*	3505 (100)
Underweight (BMI < 18.5)	161 (4.6)
Normal (BMI: 18.5–24.9)	2263 (64.6)
Pre-obesity (BMI: 25–29.9)	722 (20.6)
Obese class 1 (BMI: 30–34.9)	209 (6.0)
Obese class 2 (BMI: 35–39.9)	94 (2.7)
Obese class 3 (BMI > 40)	56 (1.6)
Mean ± SD	23.9 ± 5.0
Min-max	15.9–56.5
**number of fetus, *n* (%)**	
*n*	3654 (100)
singleton	3537 (96.8)
twin	99 (2.7)
Status post vanishing twin event	18 (0.5)

^†^ Demographic values were not available for all patients tested. For analyzed counts, refer to n in the subsections of the table. ^‡^ Samples from a gestational age below 10th week were not accepted. ^§^ Maternal indication comprises all non-medical, patient-specific reasons for NIPT, e.g., anxiety. ^¶^ First trimester combined test.

**Table 2:** Test metrics.

Variable	*n* = 3664 ^†^
**Turn around time (days)**	
*n*	1206
Mean ± SD	3 ± 1.4
Min-max	1–6
**Failure rate**	
*n*, (%)	3664 (100)
Failure exclusively after first analysis	37(1.0)
Failure exclusively after repeat analysis	5 (0.14)
**Test options**	
*n*, (%)	3662 (100)
Trisomy 21	123 (3.4)
Common trisomies 13, 18 and 21	1741 (47.5)
Common trisomies and sex chromosomal aberrations (SCA)	1023 (27.9)
All autosomes and CNVs	166 (4.5)
All autosomes, SCA and CNVs	609 (16.6)
**Reads per sample (million)**	
Mean ± SD	9.0 ± 3.4
Min-max	2.0–51.2

^†^ Test metric values were not available for all patients tested. For analyzed counts, refer to n within each subsection of the table.

**Table 3:** Clinical follow-up of positive NIPT results for common trisomies and sex chromosomal aneuploidies.

		Trisomy 21	Trisomy 18	Trisomy 13	SCA
Reported as High risk by NIPT		18	11	3	11
Clinical follow-up information available		14	8	2	5
Invasive method	Amniocentesis	7	1	1	1
	CVS	3	2	1	0
	Unspecified invasive method	0	3	0	0
	Not performed	4	2	0	4
Pregnancy outcome	Termination of pregnancy (TOP)	12	7	1	0
	Intrauterine fetal demise (IUFD)	0	0	0	1
	Pregnancy ongoing/ live birth	2	1	1	3

CVS, chorionic villus sampling; SCA, sex chromosome aneuploidies.

**Table 4:** Confirmation rate of NIPT results positive for common trisomies and sex chromosomal aneuploidies.

	Trisomy 21	Trisomy 18	Trisomy 13	SCA
True positives after amniocentesis (AC)/chorionic villi sampling (CVS)	10/10	6/6	1/2	0/5
Discordant positives ^†^	0/10	0/6	1/2	1/5
Discordant negatives ^‡^	1 ^‡^/3664	0/3664	0/3664	NA ^§^

^†^ High-risk NIPT result, which could not be confirmed cytogenetically or clinically. ^‡^ Low-risk NIPT result that was not confirmed cytogenetically or clinically due to the detection of an aneuploidy prenatally or postnatally. ^§^ Not available.

**Table 5:** Clinical follow-up for RAA- and CNV-positive NIPT cases.

	*n*	Follow-Up Information	Intrauterine Fetal Demise	Termination of Pregnancy
Rare autosomal aneuploidies (RAA)	16	10/16 ^†^	3 ^†^/10	0/10
Copy number variation (CNV)	8	8/8	2/8	2/8

^†^ One case positive due to one vanishing twin.

## Data Availability

The data that support the findings of this study are available on request from the corresponding author. The data are not publicly available due to privacy or ethical restrictions.

## References

[1] Vogel I., Petersen O.B., Christensen R., Hyett J., Lou S., Vestergaard E.M. (2018). Chromosomal Microarray as Primary Diagnostic Genomic Tool for Pregnancies at Increased Risk within a Population-Based Combined First-Trimester Screening Program: CMA for Increased Risk on CFTS. Ultrasound Obstet. Gynecol.

[2] Badeau M., Lindsay C., Blais J., Nshimyumukiza L., Takwoingi Y., Langlois S., Légaré F., Giguère Y., Turgeon A.F., Witteman W. (2017). Genomics-Based Non-Invasive Prenatal Testing for Detection of Fetal Chromosomal Aneuploidy in Pregnant Women. Cochrane Database Syst. Rev..

[3] Gil M.M., Accurti V., Santacruz B., Plana M.N., Nicolaides K.H. (2017). Analysis of Cell-Free DNA in Maternal Blood in Screening for Aneuploidies: Updated Meta-Analysis: Cell-Free DNA in Screening for Aneuploidies. Ultrasound Obstet. Gynecol..

[4] Petersen O.B., Vogel I., Ekelund C., Hyett J., Tabor A. (2013). Potential Diagnostic Consequences of Applying Noninvasive Prenatal Testing: Populationbased Study from a Country with Existing Firsttrimester Screening. Ultrasound Obstet. Gynecol..

[5] Lindquist A., Poulton A., Halliday J., Hui L. (2018). Prenatal Diagnostic Testing and Atypical Chromosome Abnormalities Following Combined First-Trimester Screening: Implications for Contingent Models of Non-Invasive Prenatal Testing. Ultrasound Obstet. Gynecol..

[6] Wellesley D., Dolk H., Boyd P.A., Greenlees R., Haeusler M., Nelen V., Garne E., Khoshnood B., Doray B., Rissmann A. (2012). Rare Chromosome Abnormalities, Prevalence and Prenatal Diagnosis Rates from Population-Based Congenital Anomaly Registers in Europe. Eur. J. Hum. Genet..

[7] Pertile M.D., Halks-Miller M., Flowers N., Barbacioru C., Kinnings S.L., Vavrek D., Seltzer W.K., Bianchi D.W. (2017). Rare Autosomal Trisomies, Revealed by Maternal Plasma DNA Sequencing, Suggest Increased Risk of Feto-Placental Disease. Sci. Transl. Med..

[8] Brison N., Neofytou M., Dehaspe L., Bayindir B., Van Den Bogaert K., Dardour L., Peeters H., Van Esch H., Van Buggenhout G., Vogels A. (2018). Predicting Fetoplacental Chromosomal Mosaicism during Non-Invasive Prenatal Testing. Prenat. Diag..

[9] Scott F., Bonifacio M., Sandow R., Ellis K., Smet M.-E., McLennan A. (2018). Rare Autosomal Trisomies: Important and Not so Rare. Prenat. Diagn..

[10] Van Opstal D., van Maarle M.C., Lichtenbelt K., Weiss M.M., Schuring-Blom H., Bhola S.L., Hoffer M.J.V., Huijsdens-van Amsterdam K., Macville M.V., Kooper A.J.A. (2017). Origin and Clinical Relevance of Chromosomal Aberrations Other than the Common Trisomies Detected by Genome-Wide NIPS: Results of the TRIDENT Study. Genet. Med..

[11] Chatron N., Till M., Abel C., Bardel C., Ramond F., Sanlaville D., Schluth-Bolard C. (2019). Detection of Rare Autosomal Trisomies through Non-invasive Prenatal Testing: Benefits for Pregnancy Management. Ultrasound Obstet. Gynecol..

[12] Lau T.K., Cheung S.W., Lo P.S.S., Pursley A.N., Chan M.K., Jiang F., Zhang H., Wang W., Jong L.F.J., Yuen O.K.C. (2014). Non-Invasive Prenatal Testing for Fetal Chromosomal Abnormalities by Low-Coverage Whole-Genome Sequencing of Maternal Plasma DNA: Review of 1982 Consecutive Cases in a Single Center: Clinical Application of NIPT. Ultrasound Obstet. Gynecol..

[13] Kleinfinger P., Lohmann L., Luscan A., Trost D., Bidat L., Debarge V., Castaigne V., Senat M.-V., Brechard M.-P., Guilbaud L. (2020). Strategy for Use of Genome-Wide Non-Invasive Prenatal Testing for Rare Autosomal Aneuploidies and Unbalanced Structural Chromosomal Anomalies. J. Clin. Med..

[14] Lefkowitz R.B., Tynan J.A., Liu T., Wu Y., Mazloom A.R., Almasri E., Hogg G., Angkachatchai V., Zhao C., Grosu D.S. (2016). Clinical Validation of a Noninvasive Prenatal Test for Genomewide Detection of Fetal Copy Number Variants. Am. J. Obstet. Gynecol..

[15] Fiorentino F., Bono S., Pizzuti F., Duca S., Polverari A., Faieta M., Baldi M., Diano L., Spinella F. (2017). The Clinical Utility of Genome-Wide Non Invasive Prenatal Screening: The Clinical Utility of Genome-Wide CfDNA Screening. Prenat. Diagn..

[16] van der Meij K.R.M., Sistermans E.A., Macville M.V.E., Stevens S.J.C., Bax C.J., Bekker M.N., Bilardo C.M., Boon E.M.J., Boter M., Diderich K.E.M. (2019). TRIDENT-2: National Implementation of Genome-Wide Non-Invasive Prenatal Testing as a First-Tier Screening Test in the Netherlands. Am. J. Hum. Genet..

[17] Liang D., Lin Y., Qiao F., Li H., Wang Y., Zhang J., Liu A., Ji X., Ma D., Jiang T. (2018). Perinatal Outcomes Following Cell-Free DNA Screening in >32,000 Women: Clinical Follow-up Data from a Single Tertiary Center. Prenat. Diagn..

[18] Huijsdens–van Amsterdam K., Straver R., van Maarle M.C., Knegt A.C., Van Opstal D., Sleutels F., Smeets D., Sistermans E.A. (2018). Mosaic Maternal 10qter Deletions Are Associated with FRA10B Expansions and May Cause False-Positive Noninvasive Prenatal Screening Results. Genet. Med..

[19] Van Den Bogaert K., Lannoo L., Brison N., Gatinois V., Baetens M., Blaumeiser B., Boemer F., Bourlard L., Bours V., De Leener A. (2021). Outcome of Publicly Funded Nationwide First-Tier Noninvasive Prenatal Screening. Genet. Med..

[20] Christiaens L., Chitty L.S., Langlois S. (2021). Current Controversies in Prenatal Diagnosis: Expanded NIPT That Includes Conditions Other than Trisomies 13, 18, and 21 Should Be Offered. Prenat. Diagn..

[21] Kim S.K., Hannum G., Geis J., Tynan J., Hogg G., Zhao C., Jensen T.J., Mazloom A.R., Oeth P., Ehrich M. (2015). Determination of Fetal DNA Fraction from the Plasma of Pregnant Women Using Sequence Read Counts: Determination of Fetal DNA Fraction from the Plasma of Pregnant Women Using Sequence Read Counts. Prenat. Diagn..

[22] Dharajiya N.G., Namba A., Horiuchi I., Miyai S., Farkas D.H., Almasri E., Saldivar J.-S., Takagi K., Kamei Y. (2015). Uterine Leiomyoma Confounding a Noninvasive Prenatal Test Result: Uterine Leiomyoma Confounding a Prenatal Test Result. Prenat. Diagn..

[23] Pertile M.D., Flowers N., Vavrek D., Andrews D., Kalista T., Craig A., Deciu C., Duenwald S., Meier K., Bhatt S. (2021). Performance of a Paired-End Sequencing-Based Noninvasive Prenatal Screening Test in the Detection of Genome-Wide Fetal Chromosomal Anomalies. Clin. Chem..

[24] Grati F. (2014). Chromosomal Mosaicism in Human Feto-Placental Development: Implications for Prenatal Diagnosis. J. Clin. Med..

[25] Robinson W.P., Barrett J., Bernard L., Telenius A., Bernasconi F., Wilson R.D., Best R.G., Howard-Peebles P.N., Langlois S., Kalousek D.K. (1997). Meiotic Origin of Trisomy in Confined Placental Mosaicism Is Correlated with Presence of Fetal Uniparental Disomy, High Levels of Trisomy in Trophoblast, and Increased Risk of Fetal Intrauterine Growth Restriction. Am. J. Hum. Genet..

[26] Toutain J., Labeau-Gauzere C., Barnetche T., Horovitz J., Saura R. (2010). Confined Placental Mosaicism and Pregnancy Outcome: A Distinction Needs to Be Made between Types 2 and 3. Prenat. Diagn..

[27] Eggermann T., Soellner L., Buiting K., Kotzot D. (2015). Mosaicism and Uniparental Disomy in Prenatal Diagnosis. Trends Mol. Med..

[28] Benn P., Malvestiti F., Grimi B., Maggi F., Simoni G., Grati F.R. (2019). Rare Autosomal Trisomies: Comparison of Detection through Cell-free DNA Analysis and Direct Chromosome Preparation of Chorionic Villus Samples. Ultrasound Obstet. Gynecol..

[29] Peng H., Yang J., Wang D., Guo F., Hou Y., Yin A. (2021). Outcomes of Pregnancies with Trisomy 16 Mosaicism Detected by NIPT: A Series of Case Reports. Mol. Cytogenet..

[30] Neiswanger K., Hohler P.M., Hively-Thomas L.B., McPherson E.W., Hogge W.A., Surti U. (2006). Variable Outcomes in Mosaic Trisomy 16: Five Case Reports and Literature Analysis. Prenat. Diagn..

[31] Rafalko J.M., Caldwell S., Tynan J., Almasri E., Weinblatt V., McCullough R. (2021). Impact of Mosaicism Ratio on Positive Predictive Value of CFDNA Screening. Prenat. Diagn..

[32] Flowers N.J., Burgess T., Giouzeppos O., Shi G., Love C.J., Hunt C.E., Scarff K.L., Archibald A.D., Pertile M.D. (2020). Genome-Wide Noninvasive Prenatal Screening for Carriers of Balanced Reciprocal Translocations. Genet. Med..

[33] Vollenhoven B.J., Lawrence A.S., Healy D.L. (1990). Uterine Fibroids: A Clinical Review. Br. J. Obstet. Gynaecol..

[34] Bowden W., Skorupski J., Kovanci E., Rajkovic A. (2009). Detection of Novel Copy Number Variants in Uterine Leiomyomas Using High-Resolution SNP Arrays. Mol. Hum. Reprod..

[35] Ishwad C.S., Ferrell R.E., Hanley K., Davare J., Meloni A.M., Sandberg A.A., Surti U. (1997). Two Discrete Regions of Deletion at 7q in Uterine Leiomyomas. Genes Chromosom. Cancer.

[36] Dharajiya N.G., Grosu D.S., Farkas D.H., McCullough R.M., Almasri E., Sun Y., Kim S.K., Jensen T.J., Saldivar J.-S., Topol E.J. (2018). Incidental Detection of Maternal Neoplasia in Noninvasive Prenatal Testing. Clin. Chem..

[37] Przybyl J., Spans L., Lum D.A., Zhu S., Vennam S., Forgó E., Varma S., Ganjoo K., Hastie T., Bowen R. (2019). Detection of Circulating Tumor DNA in Patients With Uterine Leiomyomas. JCO Precis. Oncol..

[38] Bianchi D.W., Chudova D., Sehnert A.J., Bhatt S., Murray K., Prosen T.L., Garber J.E., Wilkins-Haug L., Vora N.L., Warsof S. (2015). Noninvasive Prenatal Testing and Incidental Detection of Occult Maternal Malignancies. JAMA.

[39] Ji X., Li J., Huang Y., Sung P.-L., Yuan Y., Liu Q., Chen Y., Ju J., Zhou Y., Huang S. (2019). Identifying Occult Maternal Malignancies from 1.93 Million Pregnant Women Undergoing Noninvasive Prenatal Screening Tests. Genet. Med..

[40] Lenaerts L., Che H., Brison N., Neofytou M., Jatsenko T., Lefrère H., Maggen C., Villela D., Verheecke M., Dehaspe L. (2020). Breast Cancer Detection and Treatment Monitoring Using a Noninvasive Prenatal Testing Platform: Utility in Pregnant and Nonpregnant Populations. Clin. Chem..

[41] Lenaerts L., Brison N., Maggen C., Vancoillie L., Che H., Vandenberghe P., Dierickx D., Michaux L., Dewaele B., Neven P. (2021). Comprehensive Genome-Wide Analysis of Routine Non-Invasive Test Data Allows Cancer Prediction: A Single-Center Retrospective Analysis of over 85,000 Pregnancies. EClinicalMedicine.

